# Retraction Note: Comparison of 0.05% cyclosporine and 3% diquafosol solution for dry eye patients: a randomized, blinded, multicenter clinical trial

**DOI:** 10.1186/s12886-020-01524-8

**Published:** 2020-06-23

**Authors:** Chang Hyun Park, Hyung Keun Lee, Mee Kum Kim, Eun Chul Kim, Jae Yong Kim, Tae-im Kim, Hong Kyun Kim, Jong Suk Song, Kyung Chul Yoon, Do Hyung Lee, Tae-Young Chung, Chul Young Choi, Hyun Seung Kim

**Affiliations:** 1 Department of Ophthalmology, Yeouido St. Mary’s Hospital, College of Medicine, The Catholic University of Korea, 10, 63-ro, Yeongdeungpo-gu, Seoul, 07345 South Korea; 2 The Institute of Vision Research, Department of Ophthalmology, Gangnam Severance Hospital, Yonsei University College of Medicine, Seoul, South Korea; 3 Department of Ophthalmology, Seoul National University Hospital, Seoul National University College of Medicine, Seoul, South Korea; 4 Department of Ophthalmology, Bucheon St. Mary’s Hospital, College of Medicine, The Catholic University of Korea, Bucheon, South Korea; 5 Department of Ophthalmology, Asan Medical Center, University of Ulsan College of Medicine, Seoul, South Korea; 6 The Institute of Vision Research, Department of Ophthalmology, Severance Hospital, Yonsei University College of Medicine, Seoul, South Korea; 7 Department of Ophthalmology, Kyungpook National University School of Medicine, Daegu, South Korea; 8 Department of Ophthalmology, Korea University College of Medicine, Seoul, South Korea; 9 Department of Ophthalmology, Chonnam National University Medical School, Gwangju, South Korea; 10 Department of Ophthalmology, Ilsan Paik Hospital, Inje University College of Medicine, Goyang, South Korea; 11 Department of Ophthalmology, Samsung Medical Center, Sungkyunkwan University School of Medicine, Seoul, South Korea; 12 Department of Ophthalmology, Kangbuk Samsung Hospital, Sungkyunkwan University School of Medicine, Seoul, South Korea

**Correction to: BMC Ophthalmol (2019) 19:131**

**https://doi.org/10.1186/s12886-019-1136-8**

The editor has retracted this article [1]. In the accompanying trial registry [2] the authors stated that they conducted three separate arms of this clinical trial. The article only reports the results of two of the arms. The data and the results of this article are therefore unreliable. Additionally, there is considerable overlap with a previously published article by the same authors [3].

**All authors agree to this retraction

[1] Park, C.H., Lee, H.K., Kim, M.K. et al. Comparison of 0.05% cyclosporine and 3% diquafosol solution for dry eye patients: a randomized, blinded, multicenter clinical trial. BMC Ophthalmol 19, 131 (2019). 10.1186/s12886-019-1136-8_

[2] https://cris.nih.go.kr/cris/en/search/search_result_st01.jsp?seq=6936

[3] Park CH, Kim MK, Kim EC, Kim JY, Kim TI, Kim HK, Song JS, Yoon KC, Lee DH, Lee HK, Chung TY, Choi CY, Kim HS. Efficacy of Topical Cyclosporine Nanoemulsion 0.05% Compared with Topical Cyclosporine Emulsion 0.05% and Diquafosol 3% in Dry Eye. Korean J Ophthalmol. 2019 Aug;33(4):343–352. 10.3341/kjo.2018.0116

## References

[1] Park (2019). Comparison of 0.05% cyclosporine and 3% diquafosol solution for dry eye patients: a randomized, blinded, multicenter clinical trial. BMC Ophthalmol.

